# Compartment-resolved epithelial and myeloid programs are shared by pancreatic ductal adenocarcinoma and ischemic stroke

**DOI:** 10.3389/fcell.2026.1969947

**Published:** 2026-10-21

**Authors:** Hongyan Lu, Xiaoqi Qi, Mengzhe Zhang, Haoran Yang, Ming Zou

**Affiliations:** 1 Department of Neurology, Tianjin Medical University General Hospital, Tianjin, China; 2 School of Pharmacy, Tianjin Medical University, Tianjin, China; 3 Department of Thoracic Surgical Oncology, Shanghai Chest Hospital, Shanghai Institute of Thoracic Oncology, Shanghai Jiao Tong University School of Medicine, Shanghai, China; 4 Department of Lung Cancer Surgery, Tianjin Medical University Cancer Institute and Hospital, National Clinical Research Center for Cancer, Key Laboratory of Cancer Prevention and Therapy, Tianjin Lung Cancer Center, Tianjin, China; 5 Second Clinical Medical College, Tianjin Medical University, Tianjin, China

**Keywords:** cancer cell biology, epithelial–myeloid programs, pancreatic ductal adenocarcinoma, single-cell RNA sequencing, spatial transcriptomics, systems immunology, thrombo-inflammation, tumor microenvironment

## Abstract

**Background:**

Pancreatic ductal adenocarcinoma (PDAC) develops within a complex tumor microenvironment characterized by malignant epithelial states, myeloid inflammation, and coagulation abnormalities. Related thrombo-inflammatory transcriptional features have also been observed in ischemic stroke, but whether these diseases share reproducible molecular programs, and how such programs are organized across cellular compartments in PDAC, remains incompletely understood.

**Methods:**

We integrated transcriptomic datasets from three ischemic stroke cohorts and two paired PDAC tumor–normal cohorts using within-cohort modeling and random-effects meta-analysis. Candidate programs were subsequently investigated using independent bulk transcriptomic, single-cell RNA-sequencing, paired proteomic, spatial transcriptomic, clinical, and human genetic datasets to assess their reproducibility, cellular localization, tumor-microenvironment context, and translational relevance.

**Results:**

Iterative quality-control and replication filters identified 46 concordantly altered genes across the analyzed PDAC and ischemic stroke datasets. Six sentinel genes organized this cross-disease transcriptional signal into an epithelial program comprising CEACAM6, ANXA3, and IL1RAP; a myeloid program comprising TREM1 and IL1R2; and a coagulation-associated component represented by F5. All six genes were independently validated in PDAC, while 34 of 36 quantifiable candidate pairs showed concordant transcript–protein changes. Single-cell and spatial analyses localized the epithelial and myeloid programs to distinct malignant and immune compartments within the PDAC microenvironment. After adjustment for inferred cell abundance, the epithelial program remained the most reproducible cross-disease transcriptional feature, whereas the myeloid and coagulation-associated programs showed greater dependence on cellular composition and disease context. External stroke validation was heterogeneous, and survival, protein quantitative trait locus, and colocalization analyses did not support independent prognostic or genetic-causal interpretations.

**Conclusion:**

This multi-omic study defines a compartment-resolved cellular framework in PDAC characterized by a stable tumor-associated epithelial program and more context-dependent myeloid and coagulation-associated programs identified through cross-disease analysis with ischemic stroke. These findings provide a mechanistically informed framework for understanding cancer cell–microenvironment relationships and prioritize CEACAM6, ANXA3, IL1RAP, and TREM1 for functional investigation. They should not be interpreted as evidence that these PDAC-associated programs directly cause or mediate ischemic stroke.

## 1 Introduction

Pancreatic ductal adenocarcinoma (PDAC) has systemic consequences that extend beyond the primary tumor. In addition to its dense epithelial, stromal, and immune ecosystem, PDAC is associated with inflammation, coagulation abnormalities, and thromboembolic events. Ischemic stroke arising in patients with cancer has likewise been associated with coagulopathy and circulating features compatible with neutrophil extracellular trap (NET) formation (Navi et al., 2015; Thålin et al., 2016; Bang et al., 2019; Denorme et al., 2022). Whether these clinical phenotypes reflect a reproducible molecular program shared by the pancreatic tumor and the peripheral response to cerebral ischemia remains unknown.

Resolving such a program is not a conventional differential-expression problem. PDAC datasets profile tumor tissue, whereas most stroke datasets profile peripheral blood; concordance may therefore arise from distinct cell types, altered cellular composition, or common responses to tissue injury. Moreover, evidence that a gene changes in both diseases does not identify its cellular source or establish prognostic, therapeutic, or causal relevance. These questions require separate analyses rather than a single composite score. Single-cell studies have defined epithelial and immune heterogeneity in PDAC (Peng et al., 2019; Steele et al., 2020), and spatial studies have shown that malignant, stromal, and immune states occupy organized multicellular niches (Cui Zhou et al., 2022; Hwang et al., 2022).

Together with independent proteomic, clinical, and genetic resources, these datasets make it possible to move from cross-disease expression overlap to a compartment-resolved model and, equally importantly, to test where that model fails.

Recent oncology studies further demonstrate how multimodal data integration, external cohort validation, and interpretable computational pathology can translate molecular, radiological, and pathological measurements into clinically relevant risk stratification and outcome prediction (Ding et al., 2026a; Ding et al., 2026b; Yuan et al., 2025; Ding et al., 2026c). These studies illustrate the value of complementary data modalities and independent validation, although they address predictive modeling questions distinct from the present cross-disease molecular analysis.

We therefore analyzed each discovery cohort independently, combined standardized effects by random-effects meta-analysis, and required concordant effects, cross-disease significance, quality-control robustness, and minimum effects in every component cohort. We then interrogated retained candidates in two single-cell datasets, independent PDAC bulk data, paired CPTAC proteomics, two spatial datasets, TCGA clinical data, and pQTL–stroke analyses. This staged design resolved the shared signal into an epithelial module centered on CEACAM6, ANXA3, and IL1RAP; a myeloid program marked by TREM1 and IL1R2; and a context-dependent coagulation component represented by F5. The analysis thus defines a testable cellular architecture while keeping shared expression distinct from disease causality.

Our primary hypothesis was that PDAC and ischemic stroke share a reproducible, directionally concordant transcriptional program despite differences in tissue source, cellular composition, and measurement platform. We did not prespecify which cellular compartment would account for the most stable component of this convergence. We therefore defined three secondary questions: where the shared

Signal is expressed, whether it is reproduced in independent datasets, and whether clinical or genetic analyses provide evidence relevant to prognostic or causal interpretation. This design separated the primary test of shared expression from the secondary evaluation of its cellular source, reproducibility, and interpretive boundaries.

## 2 Materials and methods

### 2.1 Public dataset acquisition and pre-processing

No new experimental models or participants were recruited. The analysis used de-identified public human transcriptomic, proteomic, spatial, clinical, and genetic datasets. Demographic variables were included when available in the source metadata and relevant to the model. Because sex-stratified inference was not a prespecified objective and several public cohorts lacked harmonized sex metadata, sex-specific effects were not estimated. TCGA survival models included sex as a covariate. A comprehensive summary of all public datasets, genomic repositories, and analytical software tools used in this study is provided in Supplementary Table S1. Assay-specific preprocessing and quality control were performed before statistical modeling. For each bulk transcriptomic cohort, expression values were processed within the original study platform and analyzed separately rather than pooled across platforms. Probe-to-gene mapping, transformation, normalization, duplicate-probe handling, sample-level quality control, and exclusion criteria were applied on a cohort-specific basis and are summarized in Supplementary Table S1. Single-cell RNA-sequencing, spatial transcriptomic, proteomic, and TCGA datasets were likewise processed independently using modality-specific procedures described below. No expression matrices from different technological platforms were directly concatenated.

GSE16561 and GSE22255 were analyzed as stroke versus control comparisons. GSE58294 included repeated post-stroke sampling at 3, 5, and 24 h; repeated-measure structure was retained. Probes were mapped to HGNC symbols and summarized by prespecified rules. The primary meta-analysis emphasized early stroke across available compartments. Whole-blood and time-specific analyses were used as sensitivity settings.

GSE15471 and GSE28735 were analyzed using patient-matched tumor and normal pancreas. Complete expression pairs were required, yielding 35 and 45 pairs in the primary analysis. Paired linear models defined positive effects as tumor greater than normal. Exploratory correlation/PCA flags were not automatic primary exclusions. A strict-QC sensitivity retained 22 and 43 pairs. GSE62452 was not counted as an independent cohort because of sample overlap with GSE28735.

### 2.2 Study design and evidence hierarchy

The analysis followed five stages: within-disease discovery, cross-disease convergence, cellular localization, independent validation, and causal-boundary assessment. Expression matrices from different platforms were never directly concatenated. Each cohort was modeled independently, and standardized effects were combined by random-effects meta-analysis. Candidate definitions and sensitivity thresholds were fixed before downstream validation.

### 2.3 Bulk transcriptomic meta-analysis and functional profiling

Within-cohort standardized effects were integrated using restricted maximum-likelihood random-effects models. Heterogeneity was summarized using τ^2^, I^2^, and Cochran’s Q. Benjamini–Hochberg correction controlled the FDR within each analysis. Shared candidates required FDR <0.05 in both disease meta-analyses, concordant direction, replication under strict PDAC quality-control filtering, and absolute standardized effects of at least 0.3 in every component cohort.

Robustness of the PDAC signal was assessed by repeating the analysis after strict cohort-specific quality-control filtering and by examining cohort-level effect estimates for the six sentinel genes. Cell-abundance-adjusted models were additionally used to assess whether the principal candidate and module effects were sensitive to inferred cellular composition.

After identification of the cross-disease candidate set, three biologically informed candidate modules representing epithelial, myeloid/NETosis, and complement–coagulation programs were constructed. Module definitions were fixed before module-level association testing. Sample-level module scores were calculated within each cohort and analyzed independently before disease-level integration.

Rank-based functional enrichment analyses were performed using Hallmark, Reactome, Gene Ontology Biological Process, and curated NETosis, coagulation/fibrinolysis, platelet, complement, and endothelial gene sets Enrichment was evaluated within individual cohorts and integrated at the pathway level where applicable. Multiple-testing correction was performed separately within each gene-set collection.

### 2.4 Single-cell RNA sequencing data processing and cell interaction analysis

Forty-one sample archives containing 142,353 filtered cells were inspected. Quality control was performed per sample, followed by scDblFinder doublet identification. After filtering, 112,587 cells were retained. PBMC and tissue compartments were separately normalized, dimension-reduced, Harmony-integrated, embedded by UMAP, and clustered. Cell types were assigned using canonical markers, cluster-wide marker tests, and explicit review. Patient–cell-type pseudobulk profiles required at least 20 cells per unit and at least three patients per group. Differential models used the patient/sample as the replicate. Candidate detection fractions were summarized by patient and cell type. UMAP coordinates were used for display only and not as inferential replicates.

CopyKAT was run separately for each patient, with matched lymphocytes as the diploid reference. Patients were excluded from malignant epithelial comparison if the reference failed quality criteria or if fewer than 20 malignant or non-malignant epithelial cells were available. Six patients met the paired comparison threshold. Detection fractions were compared within patient. Pseudobulk differential expression used a patient-blocked edgeR model.

Candidate interactions were matched exactly to CellChatDB.human v2.1.2; substring matches were not accepted. Expression-product scores were aggregated by patient, sender group, receiver group, and interaction. A route required at least five paired patients. NicheNet was prespecified to require an FDR-supported upregulated receiver gene set. Because no such malignant epithelial set was present, formal ligand activity and target-edge inference were classified as not evaluable.

### 2.5 Spatial transcriptomic analysis

For GSE199102, quality-controlled GeoMx areas of illumination were aggregated by patient and compartment (epithelial, immune, or CAF). Paired compartment differences were tested across 19 patients and repeated in 13 untreated patients. The epithelial bridge was only partially covered because the panel included ANXA3 but not CEACAM6 or IL1RAP. GSE272362 provided gene-level spatial enrichment summaries for 30 sections. Module expression and Moran’s I were compared between primary PDAC and normal pancreas, with metastatic groups treated as extensions. The processed archive did not provide a reconstructable per-spot expression matrix; direct neighborhood and spot-level ligand–receptor analyses were not performed.

### 2.6 Multi-omics validation and clinical association analyses

GSE71729 was restricted to 145 primary PDAC and 46 normal pancreas samples. Cell lines, metastases, and unrelated normal tissues were excluded by predefined sample annotations. Differential expression used limma empirical Bayes modeling with transcriptome-wide BH correction. CRA001160 was reprocessed from the public single-cell object and summarized at patient–cell-type level using the same minimum-cell and minimum-patient principles as discovery.

Paired protein analyses used 67 matched tumor and adjacent-normal samples when the protein was quantifiable. Mean paired log2 differences, 95% confidence intervals, paired test p values, and proteome-wide FDR were calculated. Transcript–protein direction concordance was assessed among quantifiable candidates, and Spearman correlation compared PDAC transcript meta-effects with paired protein differences.

TCGA-PAAD data (Cancer Genome Atlas Research Network, 2017) were obtained through the Genomic Data Commons (GDC). GDC STAR counts were transformed to log2 (TPM +1). Primary tumors were used for purity, stage, and survival analyses. Spearman correlation tested expression against ABSOLUTE purity. Stage association used available official pathological stage groups. Cox models reported hazard ratios per one standard-deviation increase in expression and sequentially included age, sex, purity, and stage. The proportional-hazards assumption was tested for each gene and globally. BH correction was applied among candidates.

### 2.7 pQTL–stroke analyses and colocalization

Candidate pQTL and tractability information was audited using public target and pharmacology resources. For preliminary Wald screening, the strongest eligible non-palindromic cis-pQTL from the largest available protein study was matched to ischemic stroke GWAS GCST90104540. IL1R2 was tested using an independent Sun 2018 plasma pQTL. Regional colocalization used harmonized variants in GRCh38 chr2:100,992,224–102,991,047. Posterior probability for a shared causal variant (H4) was interpreted alongside H0–H3 and was not treated as binary significance.

### 2.8 Statistical analysis

Statistical units were defined as individual cohorts for meta-analyses, patients or independent samples for bulk and spatial analyses, and patient-level pseudobulks for single-cell analyses. Single cells were never treated as independent biological replicates. Unless stated otherwise, all hypothesis tests were two-sided and multiple testing was controlled using the Benjamini–Hochberg procedure with FDR <0.05. Correction was performed within predefined analytical families rather than across all analyses jointly. Transcriptome-wide differential-expression analyses were corrected across all genes tested within each cohort or meta-analysis; pathway analyses were corrected within each gene-set collection; candidate-module analyses were corrected across the tested modules within each disease and analysis setting; single-cell candidate analyses were corrected across all evaluable candidate-by-cell-subtype tests within the corresponding analysis; proteomic differential-expression results were corrected proteome-wide; and candidate-level stage, survival, spatial, and genetic analyses were corrected within their respective candidate families. All project output tables underwent programmatic validation for structural integrity and completeness. Key manuscript assertions were directly verified against raw output tables. R session configurations and software package versions were fully preserved in analytical logs.

## 3 Results

### 3.1 Sequential cross-disease filtering resolves a 46-gene shared program

We established disease-specific effects before testing convergence: three ischemic stroke cohorts were modeled independently, including one with repeated time points, and two PDAC cohorts retained their paired tumor–normal designs (Figure 1a). Among 15,221 genes tested in both meta-analyses, 230 were significant in both diseases, 126 changed in the same direction, 59 remained after strict PDAC quality-control filtering, and 46 met the minimum effect threshold in every component cohort (Figures 1b–d). Evidence coverage across eight domains separated robust overlap from orthogonal support (Figure 1e). CEACAM6, ANXA3, IL1RAP, TREM1, IL1R2, and F5 were selected as sentinels because they represented different biological compartments and evidence patterns. Their selection did not imply that all six had equivalent cellular, protein, clinical, or genetic support. The 46 genes constituted the statistically defined cross-disease candidate set. Six genes—CEACAM6, ANXA3, IL1RAP, TREM1, IL1R2, and F5—were subsequently designated as sentinel genes for focused multimodal characterization. Sentinel selection was intended to represent the major epithelial, myeloid, and coagulation-associated biological compartments and distinct orthogonal evidence patterns within the 46-gene set, rather than to define a statistically exclusive “top-six” ranking. Accordingly, the six sentinels were not assumed to have equivalent support across cellular localization, protein abundance, spatial organization, clinical association, or human genetic analyses.

**FIGURE 1:**
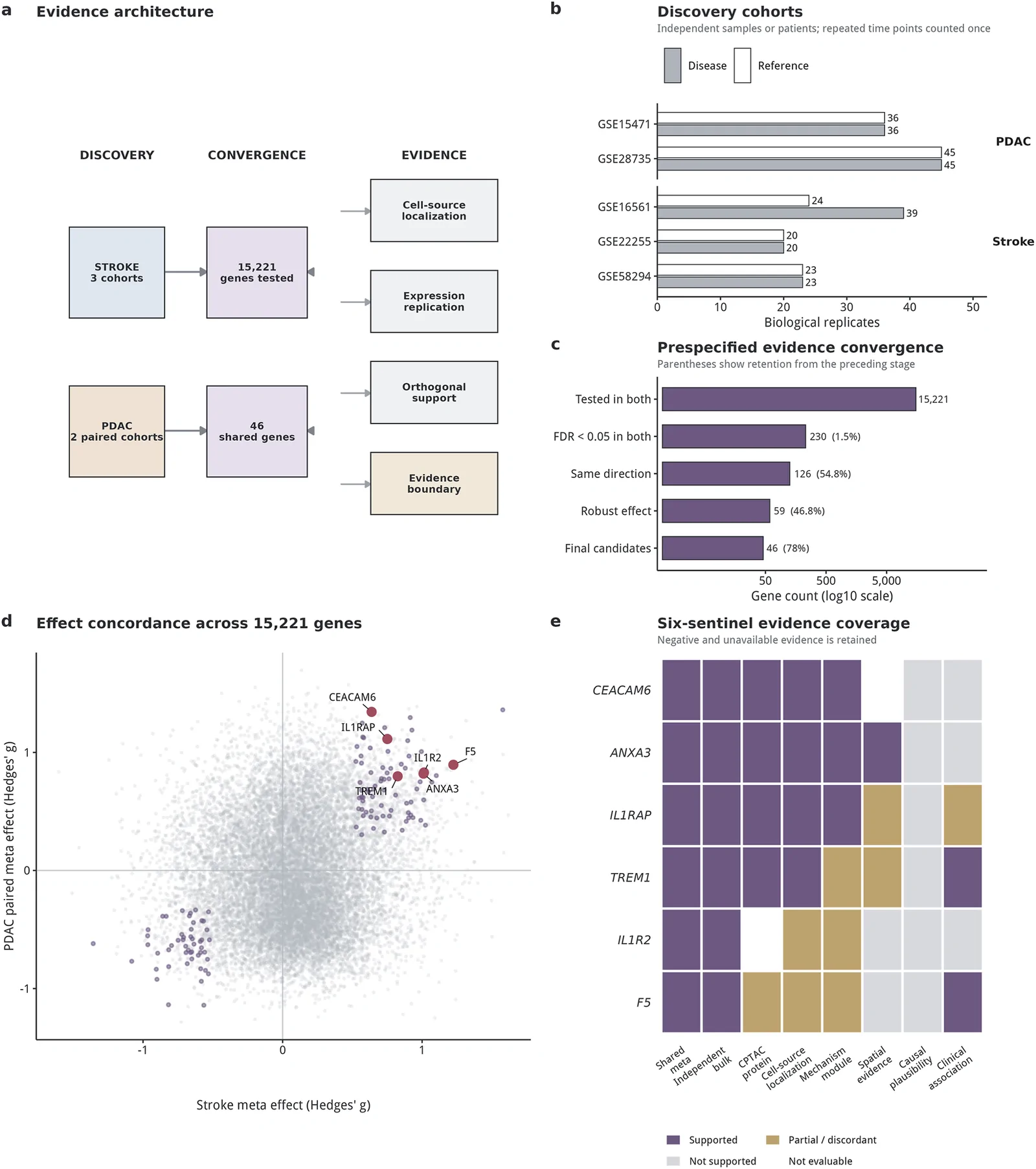
Multi-cohort and multi-omic convergence on a shared PDAC–stroke program. **(a)** Study design separating disease-specific discovery, cross-disease convergence, cell-source localization, independent validation, and causal-boundary assessment. **(b)** Cohort and sample/patient composition across discovery and validation datasets. **(c)** Stepwise filtering of 15,221 jointly tested genes to 46 robust shared candidates. **(d)** Stroke and PDAC random-effects meta-estimates for jointly tested genes; highlighted points indicate candidates passing the stated filters. **(e)** Evidence coverage for the six sentinels across eight orthogonal domains. Colors denote evidence category rather than a trained predictive score. Cohorts were modeled separately; no cross-platform expression matrices were concatenated.

### 3.2 Disease-specific meta-analyses reveal a heterogeneous stroke response and a robust PDAC program

The primary stroke meta-analysis tested 15,535 genes and identified 669 with FDR <0.05 and concordant direction; 573 also exceeded the prespecified minimum effect in every component comparison (Figures 2a–c). The number of significant concordant genes varied from 1,857 in the whole-blood early-stroke analysis to 985 and 765 in the 5-h and 24-h analyses, respectively (Figure 2d). Thus, the peripheral stroke response depended on sampling time and blood compartment rather than forming an invariant signature. Cohort-level estimates showed broadly concordant positive directions across the three stroke discovery datasets, although effect magnitude and precision varied substantially among cohorts (Supplementary Figure S3). This pattern supported distributed cross-cohort convergence while also emphasizing the heterogeneity of the peripheral stroke response.

**FIGURE 2:**
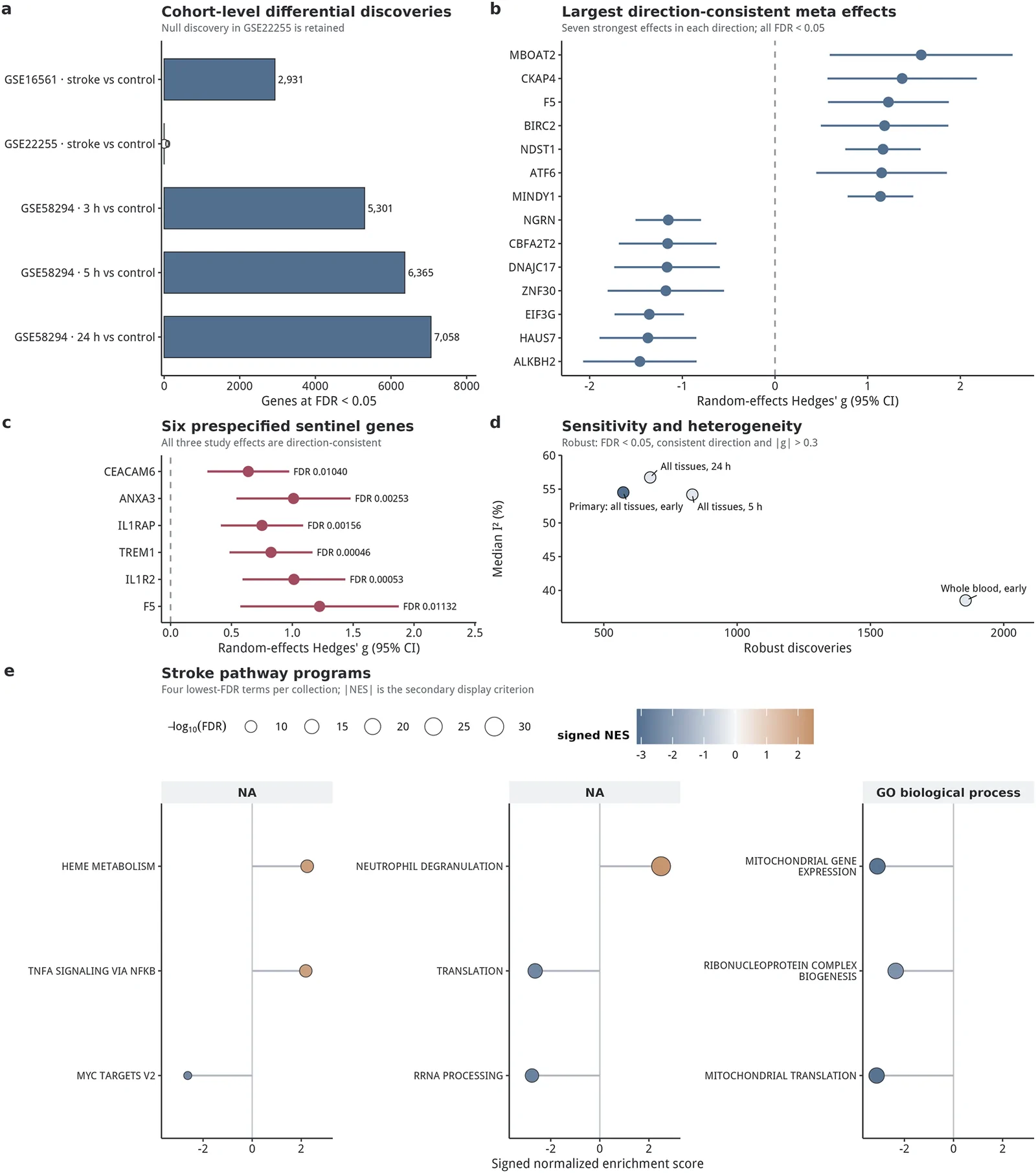
Ischemic stroke discovery identifies heterogeneous inflammatory and thrombotic programs. **(a)** Numbers of differentially expressed genes in component stroke comparisons. **(b)** Random-effects estimates and 95% confidence intervals (CIs) for leading stroke-associated genes. **(c)** Meta-effects and 95% CIs for the six sentinels. **(d)** Discovery counts and heterogeneity across primary, whole-blood, and time-specific meta-analysis scenarios. **(e)** Representative Hallmark, Reactome, Gene Ontology, and predefined mechanism enrichments. Positive effects denote higher expression in stroke. FDR was controlled by Benjamini–Hochberg correction.

Despite this heterogeneity, rank-based analyses repeatedly implicated inflammatory signaling, neutrophil activation/NETosis, coagulation and fibrinolysis, and platelet-associated programs (Figure 2e). Smaller curated complement and endothelial sets were less consistent than broader database-derived sets.

In contrast, the paired PDAC signal was broad and stable. The analysis included 35 complete tumor–normal pairs from GSE15471 and 45 from GSE28735. Among 16,938 jointly analyzed genes, 5,362 reached FDR <0.05 with concordant tumor–normal direction, and 4,639 also met the minimum within-cohort effect threshold (Figures 3a–c, e).

**FIGURE 3:**
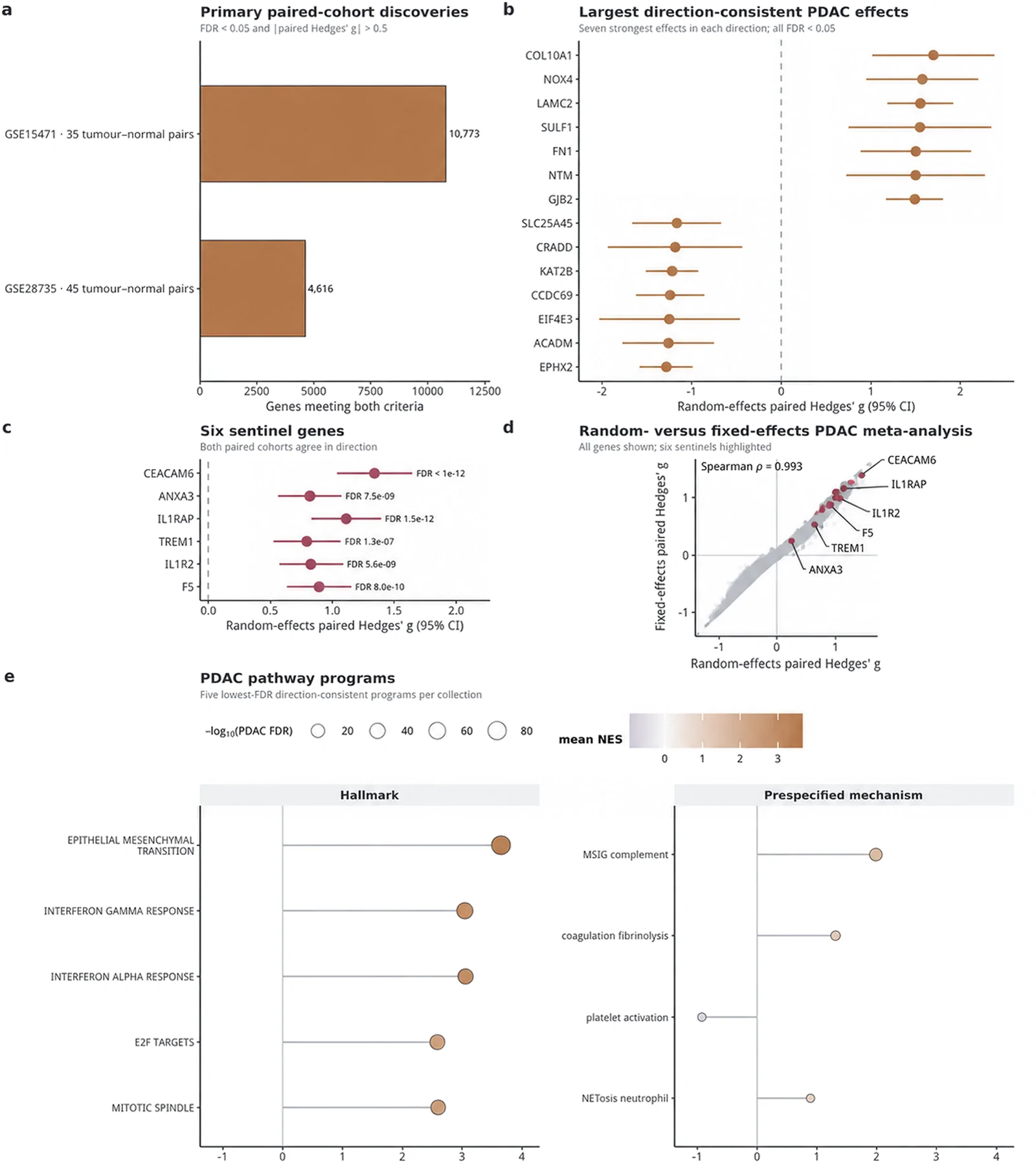
Paired PDAC analysis produces a strong and QC-robust tumor program. **(a)** Differential-expression counts in GSE15471 and GSE28735 paired tumor–normal comparisons. **(b)** Leading paired PDAC meta-effects and 95% CIs. **(c)** Six-sentinel PDAC meta-effects and 95% CIs. **(d)** Random- versus fixed-effects PDAC meta-analysis. **(e)** Representative PDAC pathway meta-analysis results. Positive effects denote higher expression in tumor. Primary analyses included 35 and 45 complete pairs; strict-QC analyses included 22 and 43 pairs.

A strict quality-control sensitivity analysis retained 22 and 43 paired samples from GSE15471 and GSE28735, respectively, and recovered 3,087 significant direction-concordant genes, including 2,942 that met the minimum effect threshold (Figure 3d; Supplementary Figure S1). Genome-wide effect estimates remained strongly aligned with the primary PDAC analysis (Spearman ρ = 0.97), and all six sentinel genes retained positive effects under strict quality control (Supplementary Figure S1e). Random- and fixed-effects estimates were closely aligned (Figure 3d).

Cohort-level forest plots further showed positive effects for all six sentinel genes in both paired PDAC discovery cohorts, although the magnitudes of individual estimates varied between datasets (Supplementary Figure S3). These analyses supported the robustness of the PDAC expression signal despite differences in effect magnitude between cohorts.

### 3.3 Cross-disease convergence separates a stable epithelial module from context-dependent downstream programs

Among the 15,221 jointly tested genes, 46 passed every cross-disease filter (Figures 4a,b). Their prioritization was not driven by statistical significance alone. We integrated effect consistency, biological annotation, secreted or membrane localization, independent expression support, cell-source localization, protein evidence, and clinical/genetic evidence (Figure 4c). The resulting candidate set contained genes associated with epithelial state, innate immunity, neutrophil biology, complement, and coagulation.

**FIGURE 4:**
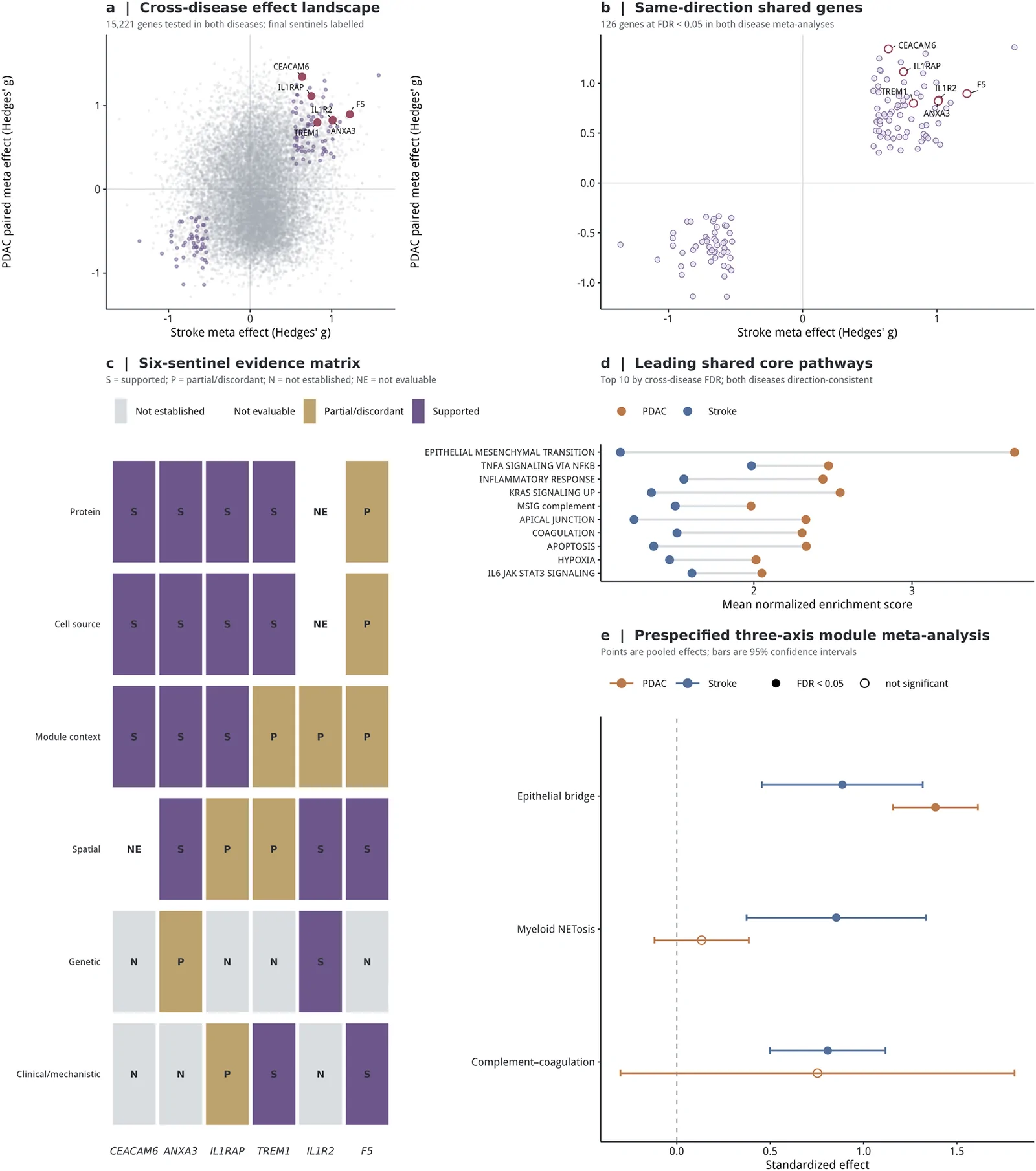
Cross-disease integration resolves a robust epithelial bridge and context-dependent downstream modules. **(a)** Stroke versus PDAC effects for all jointly tested genes. **(b)** Effects among direction-concordant, significant shared genes. **(c)** Multidomain evidence ranking of prioritized candidates. **(d)** Shared pathway normalized enrichment scores in stroke and PDAC. **(e)** Random-effects estimates for epithelial bridge, myeloid/NETosis, and complement–coagulation modules. Candidate modules were biologically defined after cross-disease candidate identification and fixed before module-level testing; error bars indicate 95% CIs.

Pathway meta-analysis across five independent comparisons identified 17 Hallmark sets and one predefined complement set that were significant and directionally concordant within both diseases. Shared programs included inflammatory/NF-κB signaling, hypoxia and vascular responses, complement–coagulation, and tissue remodeling (Figure 4d).

We next tested three biologically defined candidate modules that were constructed after identification of the cross-disease candidate set and fixed before module-level association testing. The epithelial bridge module (CEACAM6–ANXA3–IL1RAP) was the only module significant in both diseases, positive in all five discovery comparisons, and significant after MCP-counter (Becht et al., 2016) cell-abundance adjustment in each disease (Figure 4e). The myeloid/NETosis and complement–coagulation modules were prominent in stroke but did not reach within-disease FDR significance in PDAC. These results separated a robust cross-disease epithelial bridge from downstream modules that were more dependent on cellular composition and disease context (Figures 5a,b).

**FIGURE 5:**
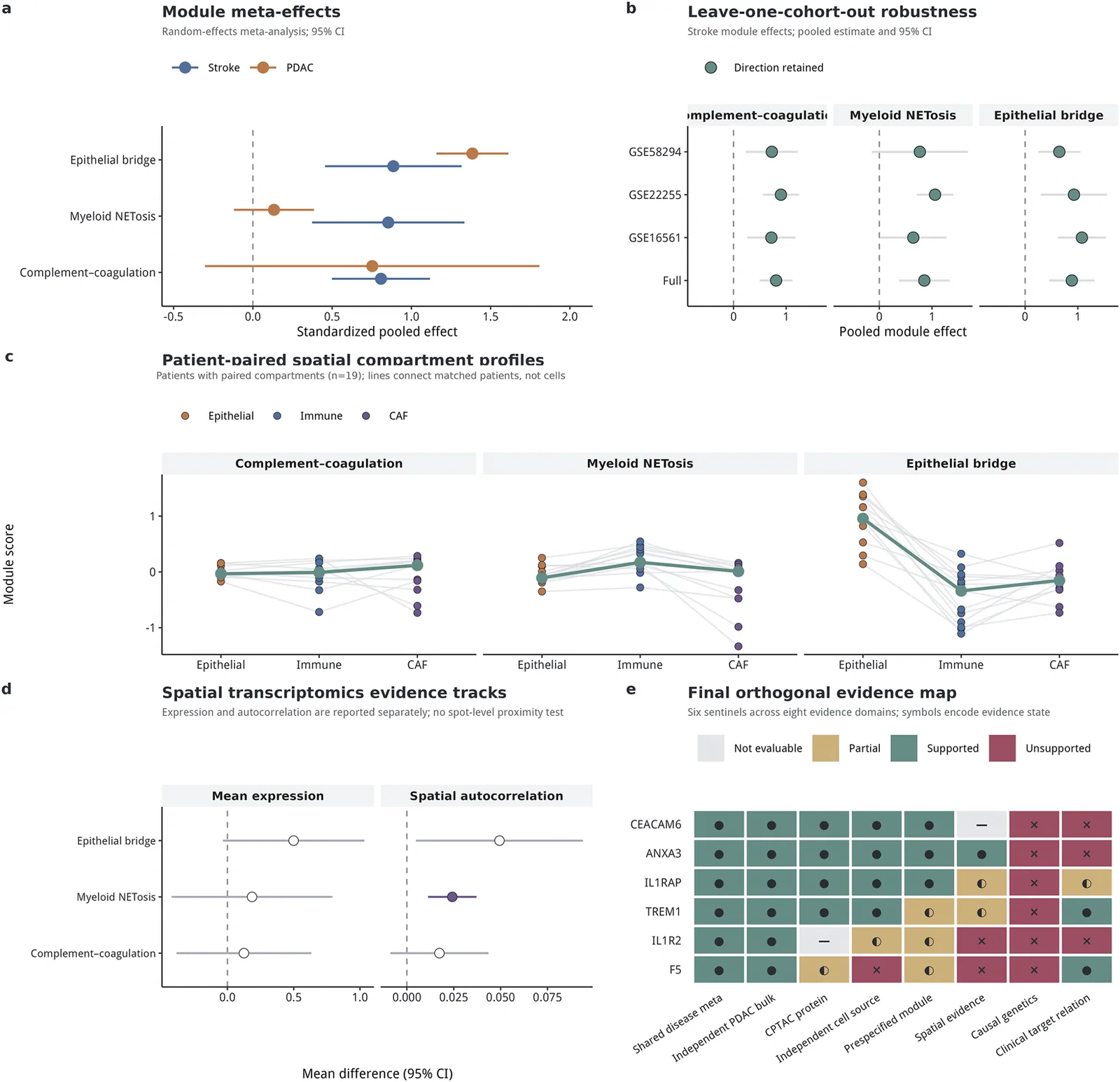
Module and spatial evidence refine the final compartmental model. **(a)** Unadjusted random-effects module estimates and 95% CIs within stroke and PDAC. **(b)** Leave-one-cohort-out robustness of the module effects. **(c)** Patient-paired GeoMx compartment profiles in 19 patients; points and 95% CIs show mean module-score differences. **(d)** Spatial-transcriptomics evidence tracks showing mean expression and spatial-autocorrelation differences between 10 primary PDAC and three normal pancreas sections. **(e)** Final categorical evidence matrix for six sentinels across eight domains. GeoMx covered ANXA3 but not CEACAM6 or IL1RAP; spatial autocorrelation does not establish direct cell proximity.

### 3.4 Independent single-cell datasets localize shared genes to epithelial and myeloid compartments

The GSE155698 resource comprised 142,353 Cell Ranger-filtered cells across PDAC tissue, adjacent pancreas, PDAC peripheral blood, and healthy peripheral blood (Steele et al., 2020). Sample-level quality control and doublet removal retained 112,587 cells: 54,277 PDAC PBMCs, 13,804 healthy PBMCs, 37,797 PDAC tissue cells, and 6,709 adjacent-tissue cells. PBMC and tissue compartments were integrated separately, yielding 22 and 28 initial clusters before marker-based refinement (Figures 6a,b).

**FIGURE 6:**
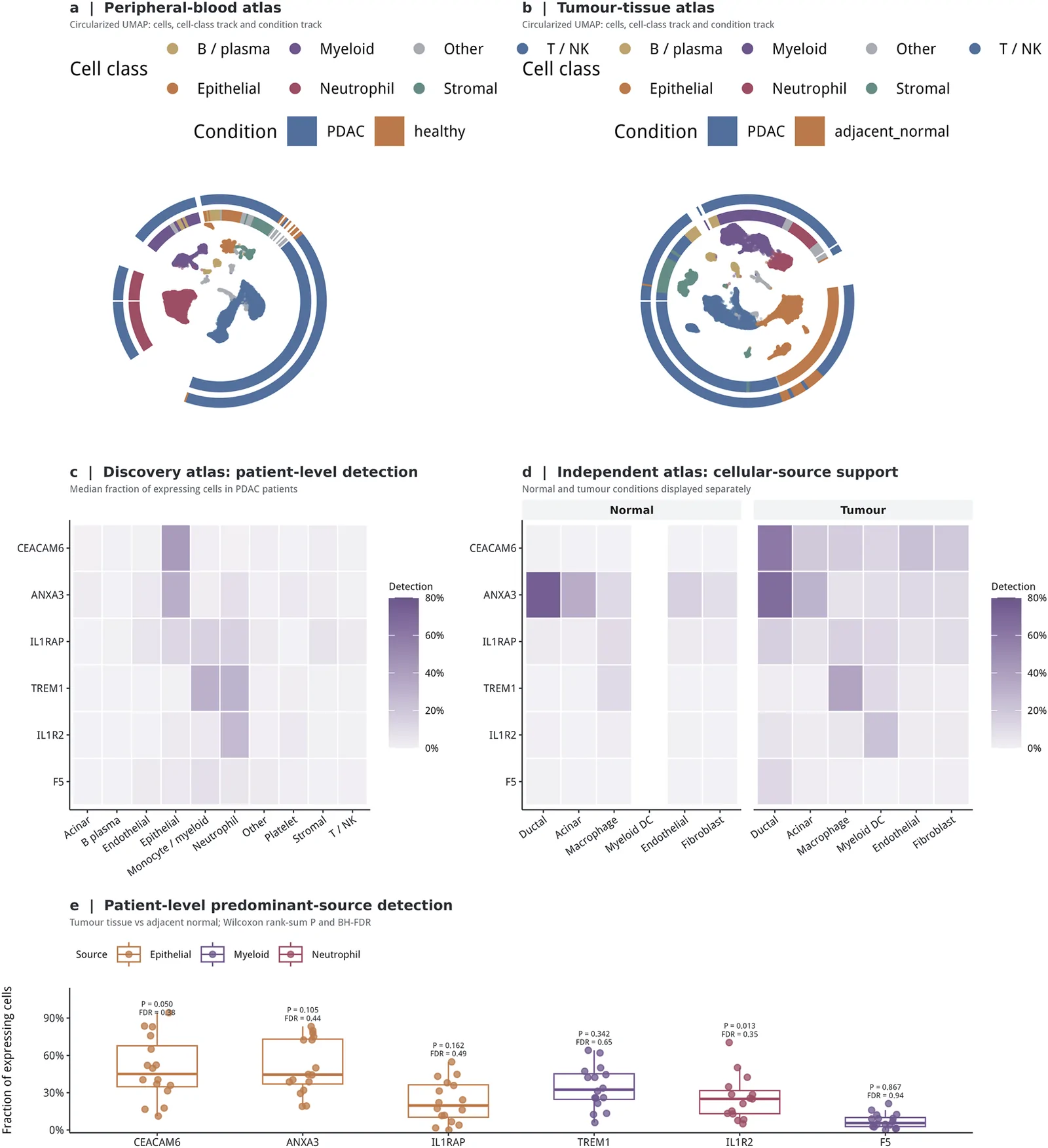
Single-cell maps separate epithelial and myeloid sources of shared genes. **(a)** Refined UMAP of PBMC compartment and **(b)** pancreatic tissue compartment from GSE155698. **(c)** Detection of six sentinels across major cell types. (d) Independent patient-level cell-source validation in CRA001160. **(e)** Patient-level detection fractions in predominant source compartments. Points are cells used for visualization; statistical inference used patient-level aggregates. GSE155698 retained 112,587 cells after QC; CRA001160 validation included 57,423 cells.

CEACAM6 localized predominantly to PDAC epithelial cells; TREM1 and IL1R2 localized to myeloid and neutrophil populations. IL1RAP and ANXA3 were detected across epithelial and granulocytic compartments, whereas F5 was detected in PBMC monocytes and tissue neutrophils (Figure 6c). Patient-level pseudobulk analysis required at least 20 cells per patient–subtype and at least three patients per group. Seventeen subtypes were evaluable, producing 100,996 gene–subtype tests and 315 tests among the 46 candidates. No candidate remained significant after global correction across all subtypes. Thus, the single-cell evidence establishes cellular source rather than a powered patient-level differential-expression claim.

Patient-by-cell-type composition analysis further showed that the principal epithelial, lymphoid, monocyte/macrophage, and neutrophil compartments were represented across multiple PDAC patients rather than being restricted to one or two dominant samples (Supplementary Figure S4). This supports interpretation of the single-cell analysis as a cellular-localization layer rather than a sample-specific clustering artifact.

To complement detection-frequency summaries, we also examined patient-level expression magnitude in the predominant source compartments. The major source patterns were also visible when mean raw counts per cell were considered alongside detection fractions, particularly for CEACAM6 and ANXA3 in epithelial populations and for TREM1 and IL1R2 in myeloid-associated compartments (Supplementary Figure S5). These analyses were descriptive and were not used to generate an additional inferential claim.

An independent reanalysis of CRA001160 included 57,423 cells from 24 PDAC and 11 normal donors (Peng et al., 2019). It reproduced ductal localization of CEACAM6 and ANXA3, myeloid localization of TREM1, and the mixed ductal–myeloid distribution of IL1RAP (Figures 6d,e). IL1R2 received partial myeloid support, while F5 showed dataset-dependent localization. The independent dataset supported the same epithelial–myeloid cellular-source architecture while identifying IL1R2 and F5 as more context dependent.

### 3.5 Patient-level analyses identify an expression-compatible epithelial–myeloid interface

Copy-number inference was performed independently within each PDAC patient using lymphocytes from the same patient as the diploid reference. Thirteen of 16 patients met the execution threshold, and 10 passed patient-level reference quality control. This identified 3,250 malignant, 977 non-malignant, and 829 unresolved epithelial cells. Six patients had at least 20 malignant and 20 non-malignant epithelial cells and entered paired analyses.

ANXA3, CEACAM6, IL1RAP, and IL1R2 generally showed higher malignant-cell detection fractions, but the leading candidate-level FDR was 0.0518. Paired detection-fraction summaries are shown in Figure 7a, with individual patient-level paired values provided in Supplementary Figure S7. We therefore interpret these paired patterns as exploratory boundary evidence rather than statistically significant malignant enrichment.

**FIGURE 7:**
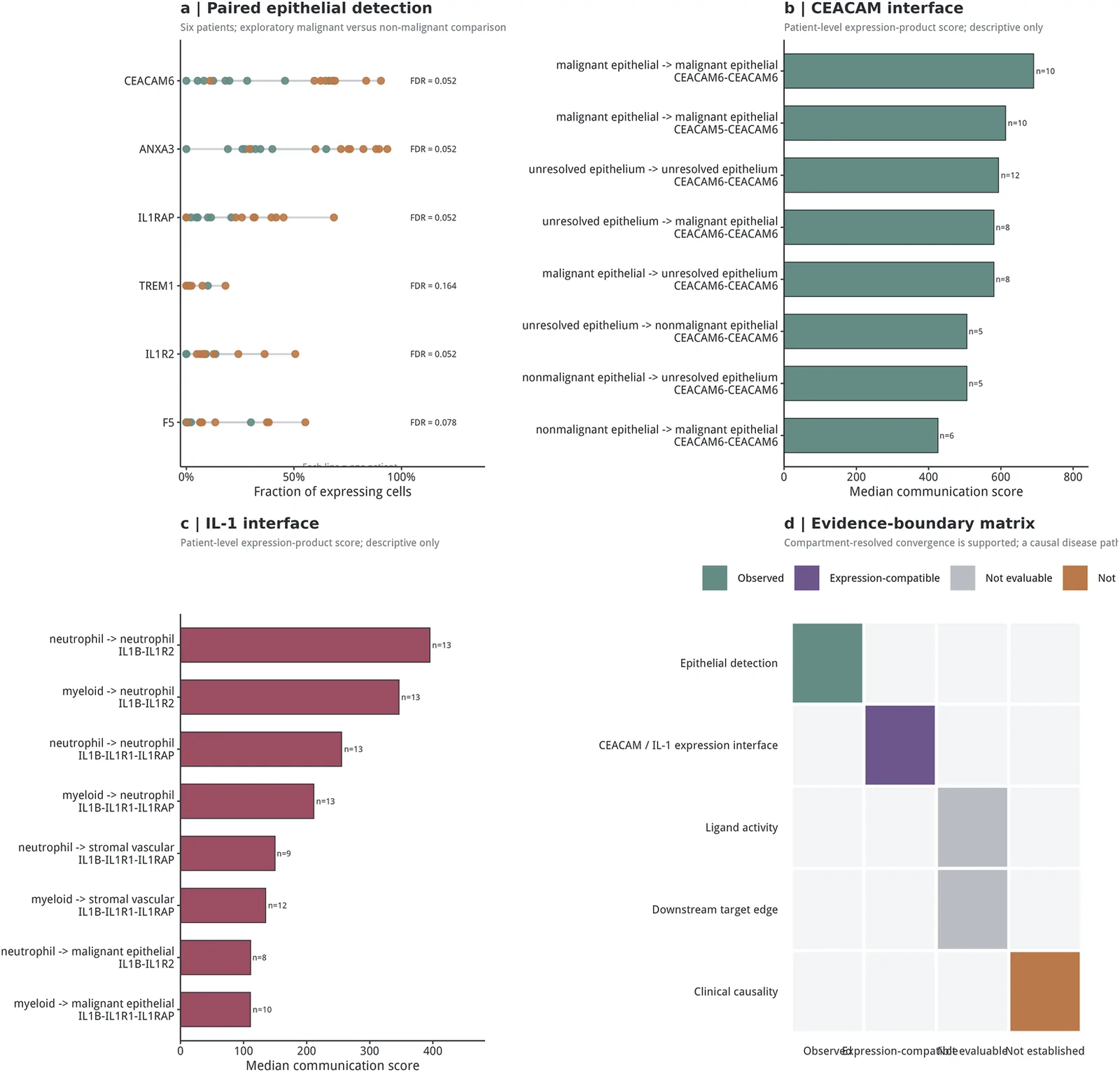
Patient-level analyses identify an expression-compatible epithelial -myeloid interface. **(a)** Paired malignant and non-malignant epithelial detection fractions in six patients; candidate-level FDR values are shown. **(b)** Patient-level CEACAM expression-product scores and **(c)** IL-1 expression-product scores; n denotes the number of evaluable paired patients for each route. These scores reflect expression compatibility only and do not demonstrate ligand secretion, receptor activation, downstream pathway engagement, or directional signaling. **(d)** Evidence-boundary matrix summarizing observed epithelial detection, expression-compatible CEACAM/IL-1 interfaces, non-evaluable ligand-activity and downstream target-edge inference, and the absence of established clinical causality. Detailed NicheNet negative results and the bounded working model are shown in Supplementary Figures S9 and S8, respectively.

Exact interrogation of CellChatDB.human identified expression-compatible CEACAM and IL-1 interaction routes involving the sentinel genes. Patient-level expression-product scores were strongest for epithelial CEACAM interfaces and for myeloid/neutrophil IL1B interactions involving IL1R1–IL1RAP or IL1R2 complexes (Figures 7b,c). These scores are descriptive measures of expression compatibility and do not demonstrate ligand secretion, receptor activation, downstream pathway engagement, or directional intercellular signaling.

Because the malignant epithelial receiver state contained no FDR-supported upregulated gene set, formal NicheNet ligand-activity and ligand–target inference were not evaluable. Six FDR-significant malignant-down genes were identified, but these did not constitute the prespecified receiver program required for formal ligand-activity analysis (Supplementary Figure S9; Figure 7d). We therefore treated the NicheNet result as an evidence-boundary analysis rather than as evidence of intercellular communication.

The resulting working model is explicitly hypothesis-generating: dashed arrows indicate putative, expression-compatible interfaces only and should not be interpreted as demonstrated signaling or causal transmission (Supplementary Figure S8).

### 3.6 Independent transcript replication and protein-level orthogonal support prioritize four core candidates

GSE71729 provided an independent bulk validation cohort of 145 primary PDACs and 46 normal pancreata (Moffitt et al., 2015). Among the 46 robust candidates, 41 were detected, 37 reached transcriptome-wide FDR <0.05, and 38 reproduced the PDAC meta-analysis direction. All six sentinels were significant and directionally replicated: CEACAM6, IL1RAP, ANXA3, TREM1, IL1R2, and F5 had log2 fold changes of 3.22, 0.56, 0.95, 0.90, 0.81, and 0.77, respectively (Figure 8a,b). Because the discovery meta-analysis reports standardized effects whereas GSE71729 is summarized using log2 fold changes, the two effect measures are not directly comparable in magnitude. Replication was therefore assessed on the basis of effect direction and statistical support rather than numerical similarity of effect size.

**FIGURE 8:**
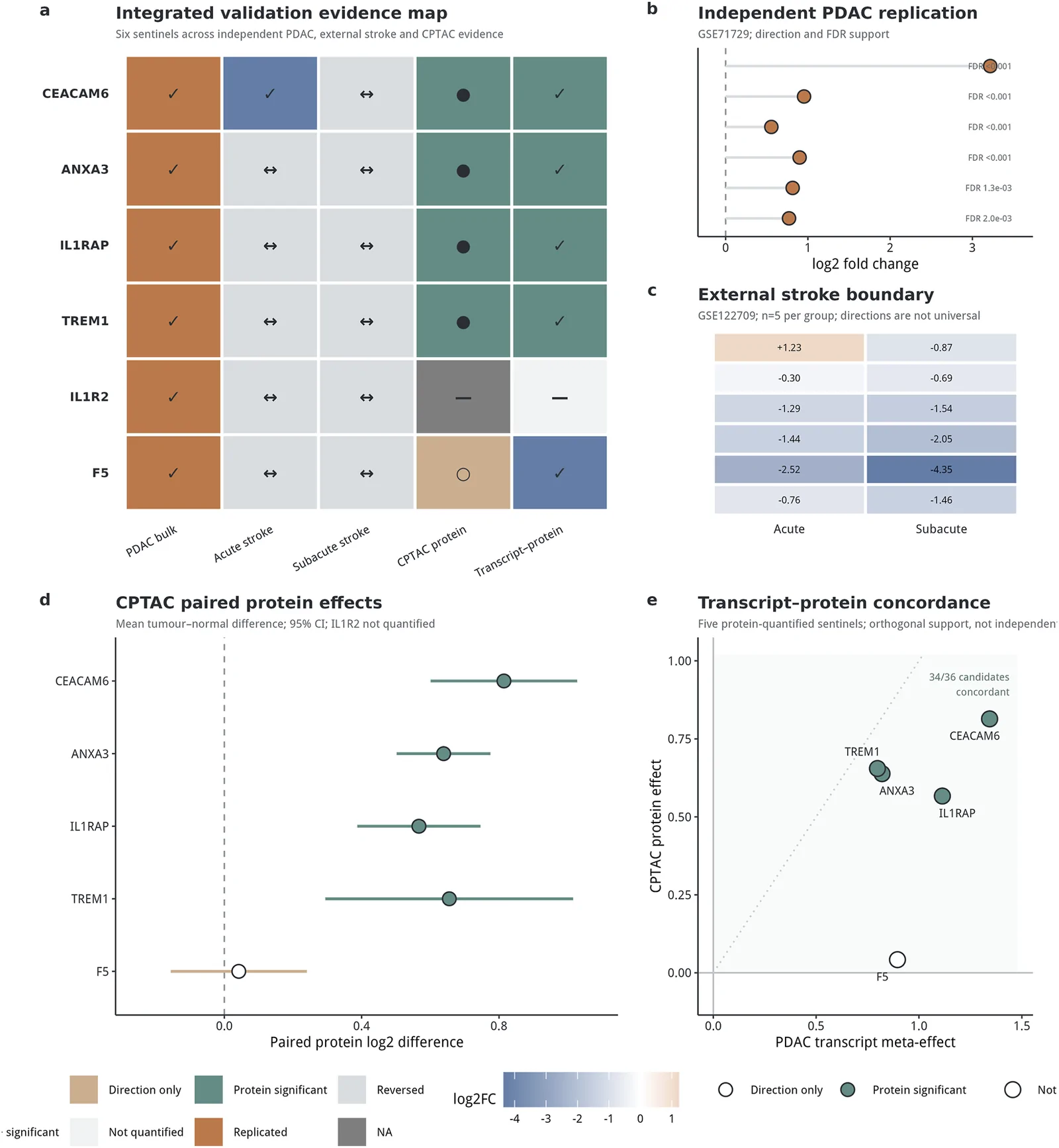
Independent transcript, external-stroke, and paired-protein validation define the evidence boundary. **(a)** Integrated validation evidence map for the six sentinels across orthogonal domains. **(b)** Independent PDAC replication in GSE71729. **(c)** External stroke boundary in GSE122709, including acute and subacute comparisons. **(d)** Mean paired CPTAC tumor-normal protein differences and 95% CIs; 21-67 pairs were available by protein. IL1R2 was not quantified. **(e)** Transcript-protein concordance for quantified sentinels. Discovery standardized effects and GSE71729 log2 fold changes are on different numerical scales and should not be compared directly in magnitude.

Paired CPTAC-PDAC proteomics included 67 matched tumor–normal pairs. Of the 46 transcript-selected candidates, 36 were quantifiable and 34 (94.4%) had concordant transcript and protein directions (binomial p = 9.71 × 10^−9^). Transcript meta-effects correlated with protein differences (Spearman ρ = 0.794, p = 7.49 × 10^−9^), but this correlation was not an independent discovery test because the candidate set was selected using transcript-level evidence. Twenty-six candidates reached proteome-wide FDR <0.05, and 25 were directionally concordant. CEACAM6, ANXA3, IL1RAP, and TREM1 showed significant positive protein effects; F5 was positive but non-significant, and IL1R2 was not quantified (Figures 8d,e). These data identified CEACAM6, ANXA3, IL1RAP, and TREM1 as the four candidates with the most consistent multi-omic support.

The independent stroke dataset addressed a different question: whether the same six genes formed a stable peripheral-blood signature across stroke settings. GSE122709 contained five acute cases, five subacute cases, and five controls. CEACAM6 was the only sentinel with a concordant acute effect; the other five were reversed, and all six were reversed in the subacute comparison (Figure 8c). Sample-level visualization confirmed that the discordant external result was not solely attributable to a single extreme observation. Individual acute, subacute, and control values showed substantial within-group variation, but the group-level directional differences remained visible for several sentinels (Supplementary Figure S6). Given the small sample size of five individuals per group, these findings were interpreted as evidence of limited transportability rather than as a definitive reversal of the discovery program. Several reversed effects were statistically significant. The cross-disease program therefore remained supported at the discovery meta-analysis level, but the six-gene subset did not behave as a universal peripheral-blood stroke classifier. Sample-level visualization showed substantial within-group variability across the five acute, five subacute, and five control samples, while the group-level directional differences remained visible for several sentinels (Supplementary Figure S6). Given the very small group sizes, these findings were interpreted as evidence of limited transportability rather than as definitive proof of a universal direction reversal.

### 3.7 Clinical and genetic analyses define evidence boundaries for translational interpretation

We next performed an evidence-boundary assessment to determine whether the shared expression signal extended to clinical prognosis or genetically supported causality TCGA-PAAD contained 178 primary tumors, four solid-tissue normal samples, and one other sample. Overall survival was evaluable for 177 patients, including 93 deaths; ABSOLUTE tumor purity was available for 149 tumors. CEACAM6 and ANXA3 correlated positively with tumor purity (ρ = 0.334, FDR = 8.92 × 10^−5^ and ρ = 0.322, FDR = 1.66 × 10^−4^, respectively). IL1R2 was also positively associated, whereas TREM1 was weakly negative and non-significant. This pattern supported epithelial localization for CEACAM6 and ANXA3 but could not distinguish a cell-state effect from a change in cell proportion.

None of the six sentinels was associated with pathological stage after FDR correction. In Cox models adjusted for age, sex, stage, and tumor purity (n = 143; 78 events), no sentinel reached FDR <0.05. Sensitivity analyses across sequential survival-model specifications likewise showed no stable independent prognostic association for any of the six sentinels (Supplementary Figure S2c). ANXA3 and IL1R2 showed evidence of proportional-hazards violation in the fully adjusted model. Thus, the TCGA analysis did not identify an independent prognostic marker and does not extend the shared expression signal to validated outcome prediction (Figures 9a–c).

**FIGURE 9:**
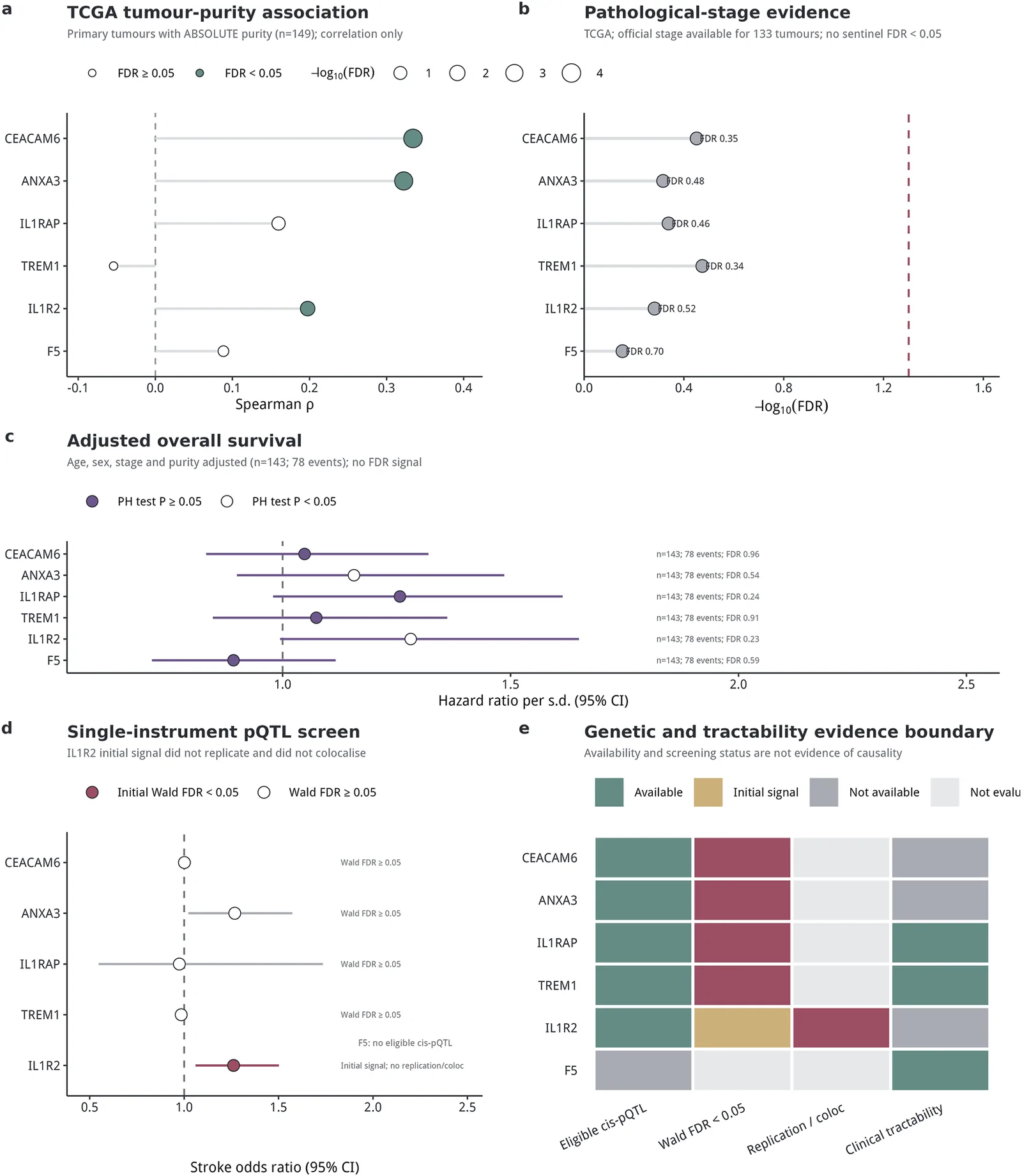
Clinical, genetic, and tractability analyses define translational scope. **(a)** Sentinel expression correlation with ABSOLUTE tumor purity in 149 TCGA-PAAD primary tumors. **(b)** Pathological-stage association in 133 tumors; dashed line denotes FDR = 0.05. **(c)** Cox hazard ratios per expression standard deviation after adjustment for age, sex, stage, and purity (n = 143; 78 events); open points flag gene-level proportional-hazards p < 0.05. **(d)** Single-instrument pQTL -stroke Wald ratios and 95% CIs. The initial IL1R2 signal did not replicate and did not colocalize; F5 had no eligible cis-pQTL. **(e)** Availability and evidence-status map. Availability or tractability does not imply causality or therapeutic efficacy.

We then assessed the genetic evidence boundary using pQTL availability, stroke associations, and regional colocalization. A single-instrument Wald screen yielded an initial signal for IL1R2 (odds ratio 1.262, FDR = 0.0442) and nominal evidence for ANXA3. The IL1R2 signal did not replicate using an independent Sun 2018 plasma pQTL. Regional colocalization across 5,026 shared variants produced PP.H4 = 0.0069 and PP.H1 = 0.9225, consistent with a strong pQTL without evidence for a shared stroke association. The posterior profile strongly favored a pQTL-only configuration over a shared causal-variant model (Supplementary Figure S2d), reinforcing the absence of genetic evidence for a shared PDAC–stroke causal mechanism. No sentinel therefore met the standard for a genetically supported causal target (Figure 9d). Tractability annotations were retained as developmental context and not as evidence that inhibiting a target would prevent stroke or benefit PDAC (Figure 9e).

### 3.8 Spatial data support epithelial and immune compartmental organization

The GeoMx analysis provided patient-level compartment contrasts across 543 quality-controlled areas of interest from 19 patients. Because the GeoMx panel covered ANXA3 but not CEACAM6 or IL1RAP, these data provide support for ANXA3-associated epithelial localization and compartmental organization rather than a complete spatial validation of the three-gene epithelial bridge. In the second spatial dataset, the processed archive provided gene-level spatial-enrichment and spatial-autocorrelation summaries but no reconstructable spot-level expression matrix. TREM1 and ANXA3 ranked highly for intratumoral spatial autocorrelation, although no sentinel remained significant after sentinel-level correction. These results support regional organization of selected candidate and myeloid/NETosis signals, but do not establish direct epithelial–myeloid proximity, ligand–receptor signaling, or functional intercellular communication.

GSE199102 GeoMx profiling yielded 543 quality-controlled areas of illumination and 19 patients with paired epithelial, immune, and CAF compartments (Hwang et al., 2022). The platform covered ANXA3 but not CEACAM6 or IL1RAP, precluding a full epithelial-module test. ANXA3/epithelial-bridge scores were higher in epithelial than immune or CAF compartments. The 14-gene NETosis module was higher in immune than epithelial compartments and immune than CAF compartments (Figure 5c). These effects persisted in 13 untreated patients. Complement–coagulation showed no stable compartment preference, and patient-level coupling between epithelial ANXA3 and immune NETosis or coagulation scores was non-significant.

GSE272362 contributed gene-level spatial enrichment statistics from 30 sections representing seven identifiable specimen groups; patient-level independence could not be reconstructed from the available metadata. The primary PDAC-versus-normal comparison therefore remains a section-level analysis, with only three normal sections. Mean module expression did not differ significantly between primary PDAC and normal pancreas. In contrast, mean Moran’s I for the 19-gene NETosis module was higher in primary PDAC (FDR = 0.0427), indicating greater within-tumor spatial organization (Figure 5d). TREM1 and ANXA3 ranked highly for intratumoral spatial autocorrelation, but no sentinel remained significant after sentinel-level correction. These results support regional organization subject to the dependence limitation and do not establish direct epithelial–myeloid proximity.

Integration of the final evidence matrix retained CEACAM6, ANXA3, IL1RAP, and TREM1 as multi-omic priority candidates and IL1R2 and F5 as context-dependent candidates (Figure 5e). Thus, independent transcript, protein, single-cell, and spatial analyses converged on four core candidates while refining the cellular roles of the remaining two.

## 4 Discussion

The principal finding of this study is that the expression program shared by PDAC and ischemic stroke is compartmentally organized rather than a uniform “thrombo-inflammatory signature.” An epithelial module was reproducible across discovery cohorts and remained significant after adjustment for inferred cell abundance, whereas NETosis and complement–coagulation modules were stronger in stroke and more dependent on disease context. This hierarchy argues for a model in which stable tumor-associated epithelial biology coexists with a variable systemic myeloid response.

CEACAM6, ANXA3, and IL1RAP define the epithelial component of this model. CEACAM6 and ANXA3 were supported by independent PDAC transcriptomes, paired proteomics, single-cell localization, and their positive relationships with tumor purity. GeoMx profiling further assigned ANXA3 to epithelial rather than immune or fibroblast compartments. Previous work has implicated CEACAM6 in pancreatic neoplasia and invasion (Duxbury et al., 2005; Blumenthal et al., 2007); here, its placement within an independently replicated cross-disease module broadens that context without implying that CEACAM6 itself mediates a cerebrovascular event. IL1RAP occupied both epithelial and immune compartments and participated in expression-compatible IL-1 receptor complexes, making it a candidate interface component rather than evidence for a unidirectional signal.

The myeloid arm was less invariant. TREM1 was the strongest myeloid anchor because its neutrophil/myeloid localization was reproduced and its PDAC transcript effect was supported at the protein level. IL1R2 and F5, in contrast, depended more strongly on dataset and modality. Spatial analyses added a further distinction: immune NETosis scores were compartment enriched, and NETosis genes showed increased spatial organization in primary PDAC without a corresponding difference in whole-section mean expression. These observations are consistent with a focal myeloid state but cannot establish direct epithelial–myeloid proximity or signaling.

Together, the discovery meta-analyses and orthogonal transcript-replication, cellular-localization, protein-support, and spatial-context analyses support a reproducible, compartment-resolved PDAC–stroke program and prioritize CEACAM6, ANXA3, IL1RAP, and TREM1.

The discordant GSE122709 result highlights the limited transportability of the six-gene peripheral-blood pattern across stroke settings. Differences in sampling time, peripheral-blood cell composition, stroke subtype or severity, systemic inflammatory state, and the very small group sizes may all contribute. Accordingly, the present study supports cross-disease convergence at the program level rather than a fixed six-gene circulating stroke classifier.

The robustness of the analysis comes from preserving cohort structure and assigning different datasets to different questions. Cohorts were modeled before meta-analysis, paired PDAC samples remained paired, quality-control exclusions were examined as sensitivity analyses, and patients—not cells—were the inferential units for single-cell comparisons. Independent bulk, protein, single-cell, spatial, clinical, and genetic analyses were used to test replication, localization, and interpretive limits rather than being collapsed into a single score.

Several gaps remain. The discovery comparison spans PDAC tissue and predominantly peripheral-blood stroke profiles, so concordance may reflect cell composition or a general response to injury rather than a tumor-derived circulating factor. No available cohort followed patients with PDAC to adjudicated ischemic stroke. Control numbers were limited in single-cell analyses, only six patients contributed to paired malignant epithelial comparisons, and the external stroke analysis included five samples per group. Communication and spatial analyses were computational, and the genetic screen was largely based on single instruments. These constraints limit inference about direction, mediation, and clinical transportability.

The next test is therefore experimental rather than another ranking exercise. Tumor-conditioned plasma or organoid secretomes could be assessed for induction of TREM1/IL1R2 states, NET formation, platelet activation, and thrombin generation in primary human systems. Perturbing CEACAM6, ANXA3, or IL1RAP while measuring these readouts would test whether the epithelial module influences the myeloid and coagulation components. Prospective PDAC cohorts with serial biospecimens, treatment exposure, thrombo-inflammatory biomarkers, and adjudicated arterial events will be needed to establish clinical relevance. Until then, CEACAM6, ANXA3, IL1RAP, and TREM1 are best viewed as experimentally prioritized components of a shared, compartment-resolved program.

## 5 Limitations

This study is limited by its reliance on public cohorts that were not specifically designed to evaluate PDAC-associated ischemic stroke. Differences in tissue compartment, sampling time, treatment exposure, cellular composition, and measurement platform may contribute to the observed cross-disease convergence. Orthogonal validation was incomplete at the six-sentinel level because not every candidate was evaluable across all proteomic and spatial resources, and the available spatial datasets did not permit definitive spot-level assessment of epithelial–myeloid proximity. Single-cell malignant-epithelial comparisons and external stroke validation were also modest in sample size. Communication and spatial analyses therefore remain computational and localization-oriented rather than functional evidence of intercellular signaling. In addition, neither the clinical nor the human genetic analyses established independent prognostic or causal effects. Functional perturbation and prospective PDAC cohorts with adjudicated cerebrovascular outcomes will be required to establish mechanistic and clinical relevance.

## 6 Availability of data and materials

All datasets analyzed in this study are publicly available from the repositories listed in Supplementary Table S1. Processed summary tables and analytical scripts supporting the present analyses are available from the corresponding author upon reasonable request and will be deposited in a public repository upon publication.

## Data Availability

The original contributions presented in the study are included in the article/Supplementary Material, further inquiries can be directed to the corresponding author.

## References

[B1] Aran D. Hu Z. Butte A. J. (2017). xCell: digitally portraying the tissue cellular heterogeneity landscape. Genome Biol. 18, 220. 10.1186/s13059-017-1349-1 29141660 PMC5688663

[B2] Bang O. Y. Chung J. W. Cho Y. H. Oh M. J. Seo W. K. Kim G. M. (2019). Circulating DNAs, a marker of neutrophil extracellular traposis and cancer-related stroke: the OASIS-cancer study. Stroke 50, 2944–2947. 10.1161/STROKEAHA.119.026373 31394991

[B3] Becht E. Giraldo N. A. Lacroix L. Buttard B. Elarouci N. Petitprez F. (2016). Estimating the population abundance of tissue-infiltrating immune and stromal cell populations using gene expression. Genome Biol. 17, 218. 10.1186/s13059-016-1070-5 27765066 PMC5073889

[B4] Blumenthal R. D. Leon E. Hansen H. J. Goldenberg D. M. (2007). Expression patterns of CEACAM5 and CEACAM6 in primary and metastatic cancers. BMC Cancer 7, 2. 10.1186/1471-2407-7-2 17201906 PMC1769503

[B5] Browaeys R. Saelens W. Saeys Y. (2020). NicheNet: modeling intercellular communication by linking ligands to target genes. Nat. Methods 17, 159–162. 10.1038/s41592-019-0667-5 31819264

[B6] Cancer Genome Atlas Research Network (2017). Integrated genomic characterization of pancreatic ductal adenocarcinoma. Cancer Cell 32, 185–203.e13. 10.1016/j.ccell.2017.07.007 28810144 PMC5964983

[B7] Cui Zhou D. Jayasinghe R. G. Chen S. Herndon J. M. Iglesia M. D. Navale P. (2022). Spatially restricted drivers and transitional cell populations cooperate with the microenvironment in untreated and chemo-resistant pancreatic cancer. Nat. Genet. 54, 1390–1405. 10.1038/s41588-022-01157-1 35995947 PMC9470535

[B8] Denorme F. Portier I. Rustad J. L. Cody M. J. de Araujo C. V. Hoki C. (2022). Neutrophil extracellular traps regulate ischemic stroke brain injury. J. Clin. Investigation 132, e154225. 10.1172/JCI154225 35358095 PMC9106355

[B9] Ding P. A. Yang S. Meng L. J. Chen S. Li S. P. Guo H. H. (2026a). A multimodal deep learning model for preoperative prediction of post-operative complications in gastric cancer. Ann. Oncol. 37, 1423–1436. 10.1016/j.annonc.2026.07.004 42463045

[B10] Ding P. Chen S. Guo H. Yang S. Wang X. Han X. (2026b). A deep learning-based digital biopsy for predicting early recurrence in gastric cancer. Nat. Commun. 17, 5220. 10.1038/s41467-026-71347-6 41986314 PMC13261139

[B11] Ding P. Yang J. Guo H. Chen S. Liu Y. Han X. (2026c). Multimodal radiopathomics model predicts postoperative metachronous liver metastasis in gastric cancer. Nat. Commun. 10.1038/s41467-026-76382-x 42834048 PMC13639083

[B12] Duxbury M. S. Matros E. Clancy T. Bailey G. Doff M. Zinner M. J. (2005). CEACAM6 is a novel biomarker in pancreatic adenocarcinoma and PanIN lesions. Ann. Surg. 241, 491–496. 10.1097/01.sla.0000154455.86404.e9 15729073 PMC1356989

[B13] Hwang W. L. Jagadeesh K. A. Guo J. A. Hoffman H. I. Yadollahpour P. Reeves J. W. (2022). Single-nucleus and spatial transcriptome profiling of pancreatic cancer identifies multicellular dynamics associated with neoadjuvant treatment. Nat. Genet. 54, 1178–1191. 10.1038/s41588-022-01134-8 35902743 PMC10290535

[B14] Jin S. Guerrero-Juarez C. F. Zhang L. Chang I. Ramos R. Kuan C. H. (2021). Inference and analysis of cell-cell communication using CellChat. Nat. Commun. 12, 1088. 10.1038/s41467-021-21246-9 33597522 PMC7889871

[B15] Moffitt R. A. Marayati R. Flate E. L. Volmar K. E. Loeza S. G. H. Hoadley K. A. (2015). Virtual microdissection identifies distinct tumor- and stroma-specific subtypes of pancreatic ductal adenocarcinoma. Nat. Genet. 47, 1168–1178. 10.1038/ng.3398 26343385 PMC4912058

[B16] Navi B. B. Reiner A. S. Kamel H. Iadecola C. Elkind M. S. V. Panageas K. S. (2015). Association between incident cancer and subsequent stroke. Ann. Neurology 77, 291–300. 10.1002/ana.24325 25472885 PMC4315703

[B17] Newman A. M. Liu C. L. Green M. R. Gentles A. J. Feng W. Xu Y. (2015). Robust enumeration of cell subsets from tissue expression profiles. Nat. Methods 12, 453–457. 10.1038/nmeth.3337 25822800 PMC4739640

[B18] Peng J. Sun B. F. Chen C. Y. Zhou J. Y. Chen Y. S. Chen H. (2019). Single-cell RNA-Seq highlights intra-tumoral heterogeneity and malignant progression in pancreatic ductal adenocarcinoma. Cell Res. 29, 725–738. 10.1038/s41422-019-0195-y 31273297 PMC6796938

[B19] Steele N. G. Carpenter E. S. Kemp S. B. Sirihorachai V. R. The S. Delrosario L. (2020). Multimodal mapping of the tumor and peripheral blood immune landscape in human pancreatic cancer. Nat. Cancer 1, 1097–1112. 10.1038/s43018-020-00121-4 34296197 PMC8294470

[B20] Thålin C. Demers M. Blomgren B. Wong S. L. von Arbin M. von Heijne A. (2016). NETosis promotes cancer-associated arterial microthrombosis presenting as ischemic stroke with troponin elevation. Thrombosis Res. 139, 56–64. 10.1016/j.thromres.2016.01.009 26916297 PMC4769435

[B21] Vittinghoff E. Glidden D. V. Shiboski S. C. McCulloch C. E. (2012). Regression Methods in Biostatistics. second edition. Springer.

[B22] Yuan W. Chen Y. Zhu B. Yang S. Zhang J. Mao N. (2025). Pancancer outcome prediction via a unified weakly supervised deep learning model. Signal Transduct. Target. Ther. 10, 285. 10.1038/s41392-025-02374-w 40897689 PMC12405520

