# Supplementary material for: Compartment-resolved epithelial and myeloid programs are shared by pancreatic ductal adenocarcinoma and ischemic stroke
Source: Front Cell Dev Biol. 2026 Oct 21;14:1969947. doi: 10.3389/fcell.2026.1969947 (PMC13731346; doi:10.3389/fcell.2026.1969947)
Supplement: Supplementary file 1 [file Table1.docx]

Supplementary Table S1. Key resources, public datasets, and analytical software packages utilized in this study.

| REAGENT or RESOURCE | SOURCE | IDENTIFIER |
| --- | --- | --- |
| Ischemic stroke expression cohort | GEO | GSE16561 |
| Ischemic stroke expression cohort | GEO | GSE22255 |
| Ischemic stroke longitudinal cohort | GEO | GSE58294 |
| External stroke PBMC RNA-seq | GEO | GSE122709 |
| Paired PDAC expression cohort | GEO | GSE15471 |
| Paired PDAC expression cohort | GEO | GSE28735 |
| Independent PDAC bulk cohort | GEO | GSE71729 |
| PDAC tissue/PBMC single-cell data | GEO | GSE155698 |
| Independent PDAC single-cell data | GSA | CRA001160 / PRJCA001063 |
| TCGA pancreatic adenocarcinoma | GDC | TCGA-PAAD |
| PDAC proteomics | CPTAC | CPTAC-PDAC |
| PDAC GeoMx spatial data | GEO | GSE199102 |
| PDAC spatial enrichment data | GEO | GSE272362 |
| Ischemic stroke GWAS | GWAS Catalog | GCST90104540 |
| CellChatDB.human | CellChat | v2.1.2 |
| R | R Foundation | v4.3.2 |
| limma | Bioconductor | v3.58.1 |
| edgeR | Bioconductor | v4.0.16 |
| metafor | CRAN | v4.4-0 |
| Seurat | CRAN | v5.0.1 |
| Harmony | CRAN/GitHub | v1.2.0 |
| scDblFinder | Bioconductor | v1.16.0 |
| CopyKAT | GitHub/CRAN | v1.1.0 |
| MCP-counter | CRAN/GitHub | v1.2.0 |
| fgsea | Bioconductor | v1.28.0 |
| CellChat | GitHub | v2.1.2 |
